# In-depth investigation of turn-around time of full blood count tests requested from a clinical haematology outpatient department in Cape Town, South Africa

**DOI:** 10.4102/ajlm.v10i1.1318

**Published:** 2021-04-29

**Authors:** Leonard Mutema, Zivanai Chapanduka, Fungai Musaigwa, Nomusa Mashigo

**Affiliations:** 1 Department of Haematological Pathology, Tygerberg Hospital, National Health Laboratory Service and Stellenbosch University, Cape Town, South Africa; 2 Department of Internal Medicine, University of KwaZulu-Natal, Durban, South Africa

**Keywords:** audit, turn-around time, full blood count, haematology clinic

## Abstract

**Background:**

The performance of laboratories can be objectively assessed using the overall turn-around time (TAT). However, TAT is defined differently by the laboratory and clinicians; therefore, it is important to determine the contribution of all the different components making up the laboratory test cycle.

**Objective:**

We carried out a retrospective analysis of the TAT of full blood count tests requested from the haematology outpatient department at Tygerberg Academic Hospital in Cape Town, South Africa, with an aim to assess laboratory performance and to identify critical steps influencing TAT.

**Methods:**

A retrospective audit was carried out, focused on the full blood count tests from the haematology outpatient department within a period of 3 months between 01 February and 30 April 2018. Data was extracted from the National Health Laboratory Service laboratory information system. The time intervals of all the phases of the test cycle were determined and total TAT and within-laboratory (intra-lab) TAT were calculated.

**Results:**

A total of 1176 tests were analysed. The total TAT median was 275 (interquartile range [IQR] 200.0–1537.7) min with the most prolonged phase being from authorisation to review by clinicians (median 114 min; IQR: 37.0–1338.5 min). The median intra-lab TAT was 55 (IQR 40–81) min and 90% of the samples were processed in the laboratory within 134 min of registration.

**Conclusion:**

Our findings showed that the intra-lab TAT was within the set internal benchmark of 3 h. Operational phases that were independent of the laboratory processes contributed the most to total TAT.

## Introduction

The increase in the number of patients accessing the public health system and the disproportionate patient to healthcare provider ratio in developing countries necessitates more efficient and cost-effective approaches to healthcare provision.^1,2^ The efficiency of healthcare providers is evaluated in part by how rapidly diagnoses are made and patients are prioritised for treatment.^3^ In addition, the rate at which laboratory results are made accessible to clinicians impacts both the patient outcome and the overall performance of a diagnostic laboratory.^4^ A prolonged turn-around time (TAT) results in delayed diagnosis and impacts the management of patients. This may lead to prolonged hospital stay and increased costs.^5,6^ The consequences of prolonged TAT are more pronounced in an emergency service setting and in outpatient departments, where a narrow window of opportunity exists to make key management decisions.^7^ Therefore, constant monitoring and improvement of the TAT in this setting is important to ensure the efficiency of laboratory services.^8^

The TAT is crucial in clinical practice and is used as a measure of the performance of laboratories.^5^ However, the TAT definition often varies between laboratories and clinicians, often resulting in unrealistic expectations.^5,6,9,10^ Total TAT is categorised into preanalytical, analytical and post-analytical stages and some define it as time from collection of the sample to when results are available for review by clinicians.^9^

Clinicians tend to view TAT as the time between requisition of a test and when they view a result.^11^ Laboratories are inclined to exclude components of the test cycle that they have no control over, such as phlebotomy and specimen transport^12,13^ because it is difficult for laboratories to address delays in these phases.^5^ Therefore, in laboratories, the TAT is usually defined as a measure of the time taken from sample registration to authorisation of results, also known as intra-lab TAT.^5^ Although this approach is not all inclusive of the phases of total TAT, it is generally accepted as the best representation of those elements of TAT that the laboratory controls.^12^

To assess the hypothesis that our laboratory performance was within set local benchmarks and comparable with the widely accepted international benchmark of completion of 90% of the sample processing within 60 min, we performed a retrospective analysis to evaluate the respective contribution of the various phases of the test cycle to the total TAT at Tygerberg Academic Hospital (TBH) in Cape Town, South Africa.^14^

## Methods

### Ethical considerations

This retrospective study was approved by the Stellenbosch University Research Ethics Committee (S18/10/232) and performed according to the Declaration of Helsinki. A waiver of consent was obtained, and patient confidentiality was maintained. Moreover, patient-level data was not accessed.

### Study design and setting

A retrospective audit was conducted over a 3-month period between 01 February and 30 April 2018 at the haematological pathology laboratory of the tertiary referral TBH in Cape Town, South Africa. The laboratory and its reception operate on a 24-h service and have three shifts: regular shift (08:00 to 16:30), evening shift (16:30 to 20:00) and night shift (20:00 to 08:00).

The laboratory provides diagnostic pathology services to regional hospitals and clinics in the Western Cape, South Africa. At TBH laboratory reception, samples go through the processes of sorting, registration and labelling before being transported to the haematology laboratory for analysis where samples are loaded into a Siemens ADVIA 2120i haematology analyser (Siemens Healthcare Diagnostics, Erlangen, Germany). This analyser has the capacity of performing 120 full blood counts (FBCs) and differentials per hour. From the haematology analyser the preliminary results are entered into the laboratory information system (LIS) at which point the results are available to clinicians as provisional results. The laboratory fast-tracks all urgent samples and aims to release the results within 3 h. Samples from the haematology clinic are treated as urgent and are colour coded and given preference over other routine samples. The LIS (InterSystems TrakCare® Lab Enterprise, Cambridge, Massachusetts, United States) tracks the processing of samples at various time points (preanalytical, analytical, and post-analytical).

The haematology clinic is housed in a separate building from the main building that houses the haematology laboratory. At this clinic, tests are requested through paper forms which are then placed alongside the samples into transporting bags and transported to the laboratory reception by porters or, rarely, by doctors or nurses. The clinic operates Monday to Friday from 07:00 to 16:00. Clinicians start seeing patients at 08:00 and most of the patient consultations are usually complete by 14:00, after which time the clinicians leave for the ward. Patients who arrive late are seen by one clinician until 16:00 when the clinic closes.

### Data collection

We retrieved and extracted data from the LIS to determine the TAT reflecting all the operational phases. Reports with two or more missing entries of the date or time points on the LIS were excluded. Full blood counts done as part of bone marrow examination requests were also excluded as they are not reported separately but as part of the full bone marrow report. Four main phases of the test cycles were identified and evaluated. The first phase is between collection of samples (phlebotomy) and registration of the same in the laboratory. This is followed by the phase between registration of samples, processing by the haematology analyser, and loading of preliminary results into the LIS. The third phase is when preliminary results are reviewed by laboratory staff and then authorised, and the final phase is from authorisation of results to when clinicians view these results. In this study, the total TAT was defined as the time taken from specimen collection to the review of the results by the requesting clinician, while the intra-laboratory TAT was defined as the time taken from specimen registration to authorisation of results after review of preliminary results by the laboratory staff. Total TAT was therefore calculated, for each sample, by adding the time taken in the four phases which are: collection to registration, registration to acquisition into LIS, acquisition into LIS to authorisation and, finally, authorisation to review by clinicians. Intra-lab TAT was calculated by adding the time taken in the two phases which are: registration to acquisition into LIS and acquisition into LIS to authorisation of results. We also carried out a sub-analysis of samples collected in the month of April. The operation phase from the collection of samples to registration was divided into two phases: transportation time (collection to receipt in the laboratory) and sorting time (from receipt in the laboratory to registration of samples). The relative contribution of these two phases were determined and compared to that of a similar study.^12^

### Statistical analysis

Data items which included sample collection, registration, acquisition to LIS, authorisation and review by clinicians were extracted from the LIS onto Microsoft® Office Excel (Redmond, Washington, United States) 2016 (version 16.0). Assessment of normality was done using the Kolmogorov-Smirnov test at *p* < 0.05 significance level.^15^ Descriptive statistics, including median, percentages and interquartile range (IQR) were used. Intra-lab and total TAT were expressed as median with IQR and time to complete 90% of tests (90% completion time).^16^ All time intervals were calculated in minutes. The chi-square test was used to compare the expected total TAT with the observed total TAT and to compare the sorting time of our laboratory with that of a study carried out at New York Presbyterian Hospital^12^; values of *p* < 0.05 were considered statistically significant. Using the percentage contribution of each phase for all 1176 samples, median percentages were calculated. The same was done for the intra-lab TAT for each of the 1176 samples. All data analyses were performed using Statistica Software version 8 (TIBCO Software Inc., Palo Alto, California, United States).

## Results

**FIGURE 1:**
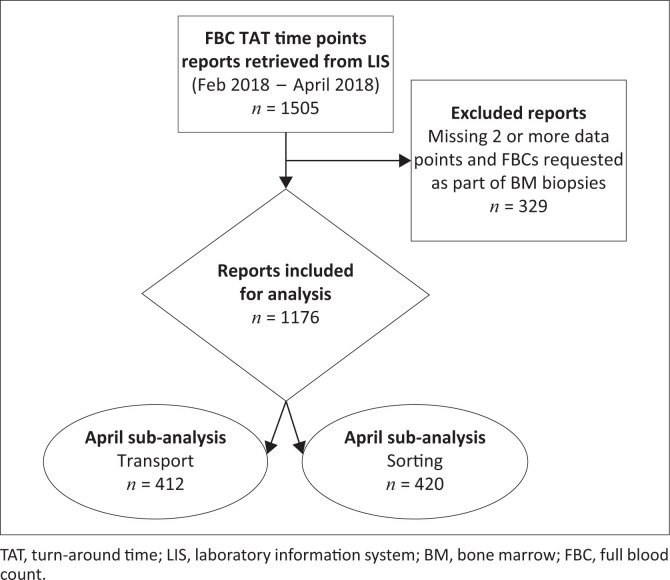
Screening and selection of reports of full blood counts requested from the haematology outpatient department at Tygerberg Academic Hospital in Cape Town, South Africa, over the period February 2018 to April 2018.

A total of 1505 FBCs were requested between 01 February and 30 April 2018 from the haematology clinic, TBH, Cape Town. We performed an analysis on a subset of the retrieved FBC reports over the study period. A total of 329 (22%) patient reports were excluded either due to missing data (2 or more missing data points) or being reports for FBCs requested as part of a bone marrow biopsy (Figure 1). In all, this retrospective study included a total of 1176 FBC reports. Delays in registration of samples after receipt by the laboratory would not be reflected on the intra-laboratory TAT but on the total TAT. Therefore, a sub-analysis of the phase between collection and registration was performed for the month of April 2018. This comprised 412 samples for the phase between collection of samples to receipt in the laboratory and 420 samples for the phase from receipt to registration. Seventy percent of the 1176 samples were collected by 9:00 and 90% by 11.00. Considering the closing time of the clinic, the clinicians had a window of 7 h and 5 h to receive results and make clinical decisions for 70% and 90% of patients.

### Intra-laboratory turn-around time and total turn-around time

The majority of the FBC samples (93.3%) had an intra-lab TAT of less than 3 h and most of them (80.1%) were processed within 90 min (Table 1). The median intra-lab TAT was 55 (IQR 40–81) min and had a median percentage of 17.1% of the total TAT (Table 2). Ninety percent of the results were ready for viewing by clinicians within 134 min of registration. The median total TAT was 275 (IQR 200.0–1537.7) min. The phases within the preanalytical and post-analytical phases contribute the most to the total TAT, with collection time to registration and time to review results by clinicians representing a median of 32.5% (IQR 7.8–48.7) and 44.3% (IQR 18.1–87.0). The total TAT was significantly delayed compared to the calculated expected TAT (*p* < 0.05).

**TABLE 1:** Contribution of the different phases of sample processing to total turn-around time and intra-lab turn-around time.

Time taken (min)	Preanalytical phase (*n* = 1176) Collection to registration (Interval 1)		Analytical phase (*n* = 1176)				Post-analytical phase (*n* = 1176) Authorisation to review by clinicians (Interval 4)		Intra-laboratory turn-around time (*n* = 1176)	
			Registration to LIS acquisition (Interval 2)		LIS acquisition to authorisation (Interval 3)					
	*n*	%	*n*	%	*n*	%	*n*	%	*n*	%
< 30	64	5.4	227	19.3	1002	85.2	267	22.7	108	9.2
30–59	114	9.7	635	54.0	100	8.5	131	11.1	551	46.9
60–89	354	30.1	198	16.8	37	3.1	122	10.4	283	24.1
90–119	271	23.0	46	3.9	11	0.9	80	6.8	99	8.4
120–149	167	14.2	7	0.6	9	0.8	57	4.8	34	2.9
150–179	49	4.2	8	0.7	2	0.2	29	2.5	21	1.8
180–209	29	2.5	8	0.7	4	0.3	20	1.7	13	1.1
210–240	26	2.2	5	0.4	2	0.2	15	1.3	4	0.3
> 240	101	8.6	41	3.5	9	0.8	454	38.6	63	5.4

Note: The times were retrieved from the National Health Laboratory Services laboratory information system at Tygerberg Academic Hospital in Cape Town, South Africa, for the period February 2018 to April 2018.

LIS, laboratory information system.

**TABLE 2:** Summary of turn-around times across phases of workflow for 1176 full blood count requests from the haematology outpatient department at Tygerberg Academic Hospital in Cape Town, South Africa, over the period February 2018 to April 2018.

Parameter	Collection to registration	Registration to LIS acquisition	LIS acquisition to authorisation	Authorisation to review of results	Intra-laboratory TAT	Total TAT
Median (minutes)	96	44	7	114	55	275
Median % of total TAT (%)	32.5	13.6	1.3	44.3	17.1	-
Median % of intra-laboratory TAT (%)	-	86.0	14.0	-	-	
Interquartile range (minutes)	73–133	33–62	2.0–19.0	37–1338.5	40–81	200–1537.7
90% completion time (minutes)	218	90	41	15530	134	15773

LIS, laboratory information system; TAT, turn-around time.

### Preanalytical phase

The preanalytical phase, defined in this study as the time from collection of samples to registration, had a median of 96 min (Table 2). Ninety percent of the samples completed this phase within 218 min. A sub-analysis of this phase during the month of April revealed a median of 60 (IQR 45–85) min for the period between collection of the sample to receipt in the laboratory (transportation) and a median of 39 (IQR 23.35–58.00) min from receipt to registration (sorting) (Table 3). Most of the samples (90%) spent less than 95 min in the sorting area. Our laboratory performed significantly worse when we compared the time spent in the sorting area for each sample in April with the median of 15 min at New York Presbyterian Hospital, another tertiary hospital described in literature, using the chi-square test.^12^

**TABLE 3:** Summary of phases between collection to registration of full blood count samples at the haematology outpatient department for the month of April 2018 at Tygerberg Academic Hospital in Cape Town, South Africa.

Parameter	Collection to receipt at laboratory	Receipt at laboratory to registration
Number of samples	412	420
Median (minutes)	60	39
Interquartile range	45–85	23–58
90% completion time (minutes)	136	95

### Analytical phases

We categorised the analytical phase into two independent intervals (interval 2 and interval 3) (Table 1). Our analysis showed that most samples (73.3%) spent less than 60 min in interval 2 while 85.2% of samples were authorised within 30 min in interval 3. Notably, a few samples, 26.6 % and 6.3%, took more than 1 h in interval 2 and interval 3. The authorisation of reports is also impacted by the battery of laboratory tests requested and this may explain the minority of samples authorised after an hour.

### Post-analytical phase

We further evaluated the time taken by clinicians to review the authorised reports (interval 4). We observed a significant number of reports (48.9%) that were accessed after 2 h, while only 22.7% of the reports were viewed within 30 min (Table 1). Notably, 38.6% of reports were viewed after 4 h. The median time for this phase was 114 (IQR 37.0–1338.5) min and the time to view 90% of the reports was 10.78 days (Table 2).

## Discussion

The constant increase in medical care cost and the growing demand for improved quality of care has necessitated the monitoring of determinants of the quality of healthcare services.^17,18^ The TAT is one of the determinants that is commonly measured, as it is both objective and easily measured.^19^ However, different views exist regarding the definition of what the composition of the TAT should be. These are often compounded by the lack of an interface for dialogue between the laboratory staff and clinicians. Howanitz et al. showed that there was also no agreement among clinicians as to the definition of TAT, with 56% using test requisition as the first step of TAT, while 44% used the time of phlebotomy as the initial step. Although most studies use reporting of results as the endpoint of TAT,^17^ this study defined total TAT as the time period from the time of phlebotomy to the time the results are reviewed by clinicians, whereas we considered the intra-laboratory TAT as the time from registration of the sample to reporting of results (authorisation). This was done in efforts to determine the relative contribution of different phases of the laboratory process to TAT and to identify phases requiring the most attention in alleviating delays. This study showed that the phases outside of the laboratory environment needed the most attention and that the laboratory was meeting its predetermined mandate.

Samples from the haematology outpatient department at TBH are regarded as urgent because for most patients, FBCs are required to make decisions on chemotherapy, transfusion and other interventions before leaving the clinic. The results of this study were therefore compared to findings of studies on TAT of emergency departments. Our findings were similar to those previously reported.^5,20^ The preanalytical and post-analytical phases accounted for a greater proportion of the total TAT, with a median of 60 min transportation time (preanalytical phase) and 48.9% of the post-analytical phase taking more than 120 min. In contrast, most of the samples were processed in the laboratory within 90 min. The median intra-lab TAT was 55 (IQR 40–81) min and 90% of samples were authorised within 134 min. Therefore, our findings suggest that the laboratory is performing within the set threshold of 3 h as stated by the TBH laboratory standard operating procedure HAE1813 for tests from the haematology clinic. This highlights the need to further interrogate the phases that the laboratory has no control over, that is, the preanalytical and post-analytical phases.

In addition to the lack of consensus on the definition of TAT, there is also no specific benchmark for TAT parameters.^21^ However, databases used to benchmark TAT thresholds can be obtained from the College of American Pathologists Q-Probes and Q-Tracks programmes.^16^ We therefore used these databases and experiences from other international hospitals to assess our performance. The 1998 College of American Pathologists Q-Probes study of emergency department TATs showed a 90% completion time for haemoglobin testing of 55 min or less.^22^ In this study 90% completion time of 60 min or less is recommended,^22^ as opposed to that of TBH which is 134 min. In a survey carried out in Nigeria involving 109 doctors, 91.3% of the respondents considered a TAT of less than 2 h as ideal for their laboratory, although their median TAT in emergency rooms was 5.12 h.^23^ In a Chinese national survey the median intra-lab TAT for white blood cells was 44.7 min.^24^ Although our laboratory performance was within the local threshold, our laboratory performed below the expectations when compared to other laboratories in a similar setting.^5,7,23,24^ The April sub-analysis exposed the sorting area as a problematic area that is responsible for prolongation of the intra-lab TAT and ultimately total TAT. Few studies focus on the analysis of this phase as the time of receipt of samples in the laboratory is often missing; therefore, the study at New York Presbyterian Hospital, which analysed total TAT with emphasis on quantification of sorting time, was best suited for comparison with this study.^12^ As with this study, sorting time was also prolonged at New York Presbyterian Hospital. Opening of sample bags, generation and printing of barcodes and decoding handwritten requests were responsible for the delays in the sorting area, which is also partly the case at TBH.^12^ Electronic requesting of tests as well as the generation of barcodes at the time of requesting tests were suggested as solutions to this delay.^12^

Total TAT was significantly prolonged compared to the expected TAT, with phases outside the laboratory causing the most delay. The time taken to view results by clinicians was the longest with a median time of 114 (IQR 37.0–1338.5) min. This could be due to the limited period during which clinicians can view results. Once this window has elapsed the results can only be viewed on the patient’s next appointment which can vary from a day to several months later. This is a limitation of total TAT in assessing laboratory performance, which explains in part why laboratories resort to the use of intra-lab TAT.^12^ These delays can be improved by ensuring patients’ punctuality and having the laboratory contact the clinicians with results as soon as they are authorised. The other cause of delay for total TAT was the time taken to transport samples from the clinic to the laboratory as well as sorting time. Porters tend to wait for samples to pile up before transporting them. Having a set time for phlebotomy and ensuring that patients avail themselves at this time may mitigate this problem. In addition, the use of pneumatic tube systems results in faster and reliable transportation of samples.^25,26^

As it is noted that a pragmatic approach is to set TAT goals locally, informed by both the published literature and by local expectations,^5^ we made the following recommendations: establishment of point-of-care testing at the haematology clinic, employment of additional staff for the sorting area or the use of an automated barcode system, and the use of pneumatic tube systems. We also recommend the use of an interactive TAT dashboard, a recently described system that offers information to enable the review of performance in real time.^27^ This may help in ensuring timely response to changes in performance. However, solutions requiring extra funding may take time to implement due to economic challenges facing the health sector in South Africa.^2,28^ As noted earlier, some results are only reviewed on the patient’s next visit and this may pose serious challenges to patient care. Therefore, the use of other communication avenues such as short message services, WhatsApp, or emails to send authorised results to clinicians is recommended. Not only will this improve the total TAT, but it will also allow clinicians to act on results immediately rather than waiting for the patient’s next appointment. Another suggestion is for clinicians to employ a clerk to review results even after the clinicians have left the clinic. For the laboratory, we recommend that considerations be made to incorporate phlebotomy and specimen transportation services into its armamentarium of service delivery. This will increase laboratory influence on preanalytical processes and subsequently improve total TAT.

### Limitations

This study was limited by the absence of an interventional process, and we therefore recommend a follow-up audit after implementing some of the proposed solutions. The results from this study cannot be extrapolated to all hospitals and departments but only to those with a similar setting, as they are solely based on the experience of the haematology clinic at TBH. The collection times were based on what was reported by the nurses at the clinic; therefore, the accuracy of the recorded time following each phlebotomy cannot be ascertained. This is not the case for other time points retrieved from the LIS as they are computer generated in real time. In addition, the laboratory receipt time for samples was not available for the entire study period. As noted earlier, 22% of the data had 2 or more missing data points which were critical in the calculation of intra-lab TAT and total TAT. This has the potential of introducing bias, which is likely if the proportion of missing data is greater than 10%.^29^ These missing data points were random occurrences on the extracted LIS data and TATs could not be computed; therefore, the samples were removed in a non-biased manner by listwise deletion. Due to the random nature of these missing points, a case is therefore made that the generalisability of the study is not affected. The statistical method of handling missing data used in this case was complete-case analysis, which only includes participants or variables that are complete on all waves of data collection.^30^

### Conclusion

This study demonstrated that TBH laboratory was compliant with its intra-lab TAT benchmark and that the 90% completion time target was achieved for samples from the haematology outpatient clinic. The phases outside the control of the laboratory were primarily responsible for prolonged total TATs. The monitoring of TAT is a powerful tool for assessing a laboratory service’s performance and contributes to patient care. However, as demonstrated in this study, monitoring and process improvement requires measurement of TATs for individual phases of the test cycle.
